# Neoantigen Fitness Model Predicts Lower Immune Recognition of Cutaneous Squamous Cell Carcinomas Than Actinic Keratoses

**DOI:** 10.3389/fimmu.2019.02799

**Published:** 2019-11-29

**Authors:** Elizabeth S. Borden, Paul Kang, Heini M. Natri, Tanya N. Phung, Melissa A. Wilson, Kenneth H. Buetow, Karen Taraszka Hastings

**Affiliations:** ^1^Department of Basic Medical Sciences, College of Medicine Phoenix, University of Arizona, Phoenix, AZ, United States; ^2^School of Life Sciences, Arizona State University, Tempe, AZ, United States; ^3^Department of Epidemiology and Biostatistics, Mel and Enid Zuckerman College of Public Health, University of Arizona, Phoenix, AZ, United States

**Keywords:** neoantigen, actinic keratosis, cutaneous squamous cell carcinoma, MHC class I, T cell receptor, cancer, immunoediting

## Abstract

A low percentage of actinic keratoses progress to develop into cutaneous squamous cell carcinoma. The immune mechanisms that successfully control or eliminate the majority of actinic keratoses and the mechanisms of immune escape by invasive squamous cell carcinoma are not well-understood. Here, we took a systematic approach to evaluate the neoantigens present in actinic keratosis and cutaneous squamous cell carcinoma specimens. We compared the number of mutations, the number of neoantigens predicted to bind MHC class I, and the number of neoantigens that are predicted to bind MHC class I and be recognized by a T cell receptor in actinic keratoses and cutaneous squamous cell carcinomas. We also considered the relative binding strengths to both MHC class I and the T cell receptor in a fitness cost model that allows for a comparison of the immune recognition potential of the neoantigens in actinic keratosis and cutaneous squamous cell carcinoma samples. The fitness cost was subsequently adjusted by the expression rates of the neoantigens to examine the role of neoantigen expression in tumor immune evasion. Our analyses indicate that, while the number of mutations and neoantigens are not significantly different between actinic keratoses and cutaneous squamous cell carcinomas, the predicted immune recognition of the neoantigen with the highest expression-adjusted fitness cost is lower for cutaneous squamous cell carcinomas compared with actinic keratoses. These findings suggest a role for the down-regulation of expression of highly immunogenic neoantigens in the immune escape of cutaneous squamous cell carcinomas. Furthermore, these findings highlight the importance of incorporating additional factors, such as the quality and expression of the neoantigens, rather than focusing solely on tumor mutational burden, in assessing immune recognition potential.

## Introduction

In the Medicare fee-for-service population, there were over one million cutaneous squamous cell carcinomas (cuSCCs) diagnosed in 2012 in the United States, and the incidence is increasing (1). While most cuSCC tumors are successfully treated with excision, ~4% of cuSCC patients develop metastases and 2% die from cuSCC (2). Especially in sun-rich states, the high incidence of cuSCC, coupled with the risk of metastasis and death, results in similar estimates of death from cuSCC as melanoma (3). Immunotherapy using immune checkpoint inhibition with the drug cemiplimab has recently been FDA-approved for the treatment of locally advanced, unresectable, and metastatic cuSCC; however, only ~50% of cuSCC patients respond to cemiplimab treatment (4). Therefore, it is critical to improve our understanding of the immune recognition of cuSCC, in order to advance the prevention and treatment options for this disease.

Actinic keratoses (AKs) are generally considered to result from cumulative ultra-violet light-induced DNA mutations, and a small percentage of these precursor lesions progress to invasive cuSCC over time (5). However, despite ongoing research, there is not yet a clear understanding of what allows some AKs to progress to cuSCC. Lesion progression involves mutations in the epithelial cells which allow malignant transformation. In addition, mutations in the tumor cells generate neoantigens which may be recognized by the naturally-occurring or therapeutically-induced immune response, and thus the immune response modulates tumor development. The interaction between cancer and the immune system is explained by the cancer immunoediting model, which has three phases (6). In the elimination phase, the immune system destroys the developing tumor before the tumor becomes clinically apparent. The elimination phase can result in complete elimination or residual cancer cell variants that resist elimination and enter the equilibrium phase. In the equilibrium phase, the immune response controls tumor growth. Editing of the immunogenicity of the tumor occurs in the equilibrium phase as a consequence of selective pressure from the immune response. Tumor cell variants that are no longer recognized by the immune system enter the escape phase and manifest as clinically apparent tumors. Examples of immune escape include the loss of tumor antigens or the loss of the ability to present the tumor antigens on MHC class I. The immunoediting process has recently been demonstrated in early stage, untreated non-small cell lung cancer (7). Tumors with intact MHC class I had a significant decrease in expressed neoantigens compared with non-neoantigenic, somatic mutations, and only tumors with intact MHC class I and immune cell infiltration exhibited a decrease in expressed neoantigens (7).

Cancer immunoediting is also observed in response to therapeutically-induced immune responses from immunotherapy with immune checkpoint inhibitors. Immune checkpoint receptors are expressed on T cells after activation and function as part of a homeostatic mechanism to turn off T cell responses (8). While immune checkpoint receptor-ligand interactions are helpful in constricting T cell responses after an infection is cleared, some tumors co-opt this mechanism to avoid eliciting a T cell response. Immune checkpoint inhibition therapy blocks this inhibitory signal and improves immune-mediated tumor destruction. Several studies have demonstrated an association of higher tumor mutational burden with improved response to immune checkpoint inhibition in melanoma and non-small cell lung cancer (9–13). A subset of these studies also analyzed the number of neoantigens and found that the number of neoantigens was also positively correlated with the response to immunotherapy (9, 11–13). However, these results have not been consistent across all cancers. Multiple myeloma patients were found to have an inverse relationship between progression-free survival and the tumor mutational burden and neoantigens (14). These opposing results suggest that there are likely additional factors influencing the relationship between neoantigens and the response to immunotherapy.

One possible factor suggested by McGranahan et al. is the homogeneity of the tumor neoantigens. Their group found that in lung cancers, in addition to the number of neoantigens, the degree of homogeneity of the tumor was highly associated with the survival of the patient (9). They found that the more homogenous the neoantigen presence was in the tumor, the greater the patient survival (9). Another factor is defined by Łuksza et al. as the “fitness cost of the tumor,” which is directly proportional to MHC class I binding and T-cell receptor (TCR) recognition potential (15). An increased fitness cost was found to highly correlate with improved response to immunotherapies, suggesting that, in addition to the number of mutations and neoantigens, the strength of these neoantigens is important in predicting the immune response (15). This finding is corroborated by Anagnostou et al. who demonstrated the changes in the neoantigens found in tumors before and after immunotherapy (16). In this study, the overall number of neoantigens increased from the original tumor to the post-treatment tumor; however, the strength of retained and gained neoantigens was less than the strength of the neoantigens that were lost (16). These results again suggest that the ability of a tumor to grow in the face of a competent immune system requires changes not only in the number of neoantigens but in their quality.

There have been a few analyses of the differences in tumor mutational burden between pre-cancerous and cancerous lesions (17–19). Two of these analyses compared the tumor mutational burden between Barrett's esophagus and esophageal adenocarcinomas and found that the tumor mutational burden increased from the pre-cancerous to the cancerous lesion in paired samples (17, 18). A single study investigated the tumor mutational burden in AKs and cuSCCs (19), but, to our knowledge, there are no reports of the number of neoantigens or the fitness costs of these neoantigens in AK or cuSCC. In order to address these gaps, we have compared the neoantigen burden and fitness cost between AKs and cuSCCs. Understanding what allows the immune system to keep AKs in equilibrium while it fails to do so with cuSCCs may help to explain which AKs progress to cuSCCs.

## Methods

### Datasets

Whole exome sequencing (WES) data with an average coverage of 135 × ± 22 (mean ± s.d.) (Illumina Hi-Seq) and RNAseq data average of 64 million reads per sample (Illumina Hi-Seq) from Chitsazzadeh et al. was used for this analysis (19). Datasets from 7 AK samples and 7 cuSCC samples from 9 different patients were kindly provided by Dr. Ken Tsai and Dr. Kimal Pajapakshe. Eight out of the 9 patients also had a normal skin sample which was used for comparison. For the remaining patient (patient 12), a saliva sample was used in place of the normal skin. These samples and the patients they come from are summarized in Table 1. Note that the AK and cuSCC samples from an individual patient are separate lesions.

**Table 1:** Summary of the samples available from each patient.

**Patient ID**	**1**	**2**	**3**	**4**	**5**	**6**	**8**	**10**	**12**
WES normal skin	X	X	X	X	X	X	X	X	
WES saliva	x			x	x	x	x	x	X
WES AK	X	X	X	X	X			X	X
WES cuSCC		X	X	X, X	X	X	X		
RNAseq normal skin		x	x	x	x	x	x	x	
RNAseq AK	x	X	X	x	X	x	x	x	x
RNAseq cuSCC		X	X	x, x	X	X	x	x	x

*X denotes any samples used in our analyses, and x denotes samples available that were not used*.

### Neoepitope Prediction

WES and RNAseq data files for normal skin, AK, and cuSCC samples were obtained as FASTQ files. WES and RNAseq FASTQ files underwent quality control using FastQC (20) and were trimmed for quality using Trimmomatic (21) IlluminaClip with the following parameters: seed_mismatches = 2, palindrome_clip_threshold = 30, simple_clip_threshold = 10, leading = 10, trailing = 10, winsize = 4, winqual = 15. Trimmed WES reads were mapped to the GRCh38.p7 reference genome using HISAT2 v. 2.1.0 (22). SAM files were converted to BAM and coordinate sorted using SAMtools v. 1.4 (23). WES BAM files were processed according to Broad Institute GATK (Genome Analysis Toolkit) best practices (24–26). Read groups were added with Picard Toolkit's AddOrReplaceReadGroups and optical duplicates marked with Picard Toolkit's MarkDuplicates (v.2.18.1, http://broadinstitute.github.io/picard/). Base quality scores were recalibrated with GATK (v.4.0.3.0) BaseRecalibrator. The BAM files were then converted to pileup format using Samtools 1.3.1 (23). To determine the neoantigens present in the AKs compared to the cuSCCs, first, high confidence single nucleotide polymorphisms (SNPs) and indels were called using VarScan2 version 2.3.9 with minimum coverage of 10, minimum variant allele frequency of 0.08, and somatic *p*-value of 0.05 (27). Somatic mutations were separated from those SNPs that fell within 1 bp of an indel position, as these were likely to be false positives due to alignment errors. The variants were annotated using the Variant Effect Predictor tool from Ensembl version 90.9 (28, 29). Finally, peptides were identified and prioritized from these variants using pVACtools version 3.0.5 (30, 31). For every variation in amino acid, all possible peptides were generated in which the changed amino acid was included at every position in a sequence. Sequences of 21 amino acids were considered. The non-mutated sequence corresponding to each of these possible neoepitopes was also extracted for comparison sake. These steps were done as outlined in the EpitopeHunter pipeline from Narang et al. (32).

### HLA Typing

HLA typing was completed for three major MHC class I genes (HLA-A, -B, and -C) using POLYSOLVER (POLYmorphic loci reSOLVER) version 1.0 (33). POLYSOLVER aligns reads from the WES data in the HLA region of each sample and then aligns these regions to a library of all known HLA alleles. Then, a Bayesian classification approach is used to determine the two alleles for each gene for each patient.

### Predicting MHC Class I Binding Epitopes

Prediction of the potential epitopes that would effectively bind to MHC class I was completed with the NetMHCpan server version 4.0 (34). Neoantigens of only 9 amino acids were considered. Scores were calculated for both the mutated peptides and their wild-type counterparts. Each was scored based on its dissociation constant to each of the alleles predicted by POLYSOLVER. The top binding potential for each neoantigen was selected independently for the wild-type and mutant peptides. These binding probabilities were then used to determine an “amplitude” (A) using the methods outlined by Łuksza et al. (15). Only neoantigens with a maximum dissociation constant of 500 nM were considered, and the ratio of the dissociation constants (K_d_) for the wild-type (WT) compared to the mutant (MT) peptides was calculated as shown here:

$$\begin{array}{l} A=K_{d}^{WT}/K_{d}^{MT} \end{array}$$

For wild-type peptides with exceptionally high predicted dissociation constants, an adjustment is made to account for the lack of accuracy of netMHCpan at predicting dissociation constants above the range in which it was trained. This adjustment is described in detail in the paper of Łuksza et al. and will therefore not be discussed further here (15).

### Predicting Neoantigen TCR Recognition

Prediction of TCR recognition potential, R, was calculated as described by Łuksza et al. (15). A BLOSUM62 similarity matrix was used to assess the sequence similarity between a neoantigen and peptide sequences that are known T cell antigens from the Immune Epitope Database (IEDB) (35). The same set of IEDB sequences was used as was optimized by Łuksza et al. for evaluation of neoantigens in melanoma and small cell lung cancer (15). The sequence similarity was then used in place of binding energies, and the TCR recognition potential was calculated as:

$$\begin{array}{l} R={Z\left(k\right)}^{-1}\underset{e\in IEDB}{\sum }\exp\left[-k\left(a-|s,e|\right)\right] \end{array}$$

Where |s,e| is the sequence similarity, a is the horizontal displacement of the binding curve, and k sets the steepness of the curve at a. Based on the model fit by Łuksza et al., the parameters a and k were set to 26 and 4.87, respectively (15). These parameters were optimized for both melanoma and lung cancer patients and shown to have consistent predictive value in both cancers. This gives us confidence that they can likewise be used with predictive value for cuSCC without the need to introduce a new training set. Finally, Z(k) is the partition function over the unbound state and all bound states, calculated as follows:

$$\begin{array}{l} Z\left(k\right)=1+\underset{e\in IEDB}{\sum }\exp\left[-k\left(a-|s,e|\right)\right] \end{array}$$

### Fitness Cost Calculation

Given the TCR recognition potential (R) and the MHC-binding amplitude (A), the fitness cost (F) of the neoantigens was calculated as described by Łuksza et al. (15):

$$\begin{array}{l} F=A\times R \end{array}$$

Rather than taking the negative of this value as was done by Łuksza et al., we have left this value as a positive and will refer to it as the fitness cost. The greater the fitness cost, the more immune recognition and immune-mediated destruction we predict for the tumor. We have chosen to look at both the average and the maximum fitness cost for the neoantigens in each tumor to avoid presupposing that the single strongest neoantigen is of sole importance in the tumor recognizability.

Recognizing that to be visible to the immune system, neoantigens must be expressed, the fitness cost was then adjusted for the magnitude of expression of the given neoantigen. Transcriptome assembly and read count quantifications were completed with Salmon version 0.11.3 (36) normalized to reads per kilobase of transcript per million mapped reads (RPKM). Using RNAseq data, the expression of each neoantigen with non-zero fitness cost was calculated as a fraction of the total read counts for these neoantigens. This fraction was multiplied by the amplitude and TCR recognition potential to generate an adjusted fitness cost.

### Statistical Analysis

Statistical Analyses were performed using STATA version 14 (STATAcorp; College Station, TX). *P*-value calculations were done using a Wilcoxon Rank Sum calculation. Correlation between variables were calculated as Spearman's Rho correlation coefficients.

### HLA Mutations and Expression

To determine the role of HLA mutations and expression on the neoantigen profile of the tumor samples, somatic mutations were identified within the coding regions for HLA-A, B, and C. The mutations identified were then compared to the regions encoding the peptide binding groove. The regions encoding the peptide binding groove are found on exons 2 and 3. HLA coding regions and peptide binding groove regions were determined by the NCBI Gene database (37). MHC class I pathway members, TAP1, TAP2, or B2M were also interrogated for mutations. RPKM-normalized RNAseq expression levels of HLA-A, B, and C as well as TAP1, TAP2, and B2M were fit with a linear regression against the adjusted maximum fitness cost. The mutation's effect on the expression of the gene was evaluated by calculating the average expression and the standard deviation of the specific gene across all samples. For the mutations found in HLA-B for patient 2 cuSCC and in HLA-C for patient 5 AK, the expression value was compared to the average expression of the respective HLA gene. A value within two standard deviations from the mean was considered unaffected by the mutation.

### Cell Enrichment Type Analysis

We used the xCell web service to deconvolute the diverse cell populations present in the cuSCC and AK samples (38). This program uses a gene signature-based method to estimate the proportions of different cell types in a bulk-RNA-sequence sample. RPKM gene expression data was used to estimate the fraction of the tumor sample made up by different cell types. In this work, the specific focus was on the immune-related cell types. Linear regressions were fit to the adjusted maximum fitness cost against the expression level of each of the following: T cell subsets (CD4 T cells, CD8 T cells, CD8 central memory T cells, CD8 effector memory T cells, CD8 naïve T cells, regulatory T cells), dendritic cell populations (immature DCs and conventional DCs), and natural killer (NK) cells. A Bonferroni correction was completed on the *p*-values from these correlations to adjust for the number of comparisons.

## Results

### Analysis of Mutation and Neoantigen Counts

**Figure 1:**
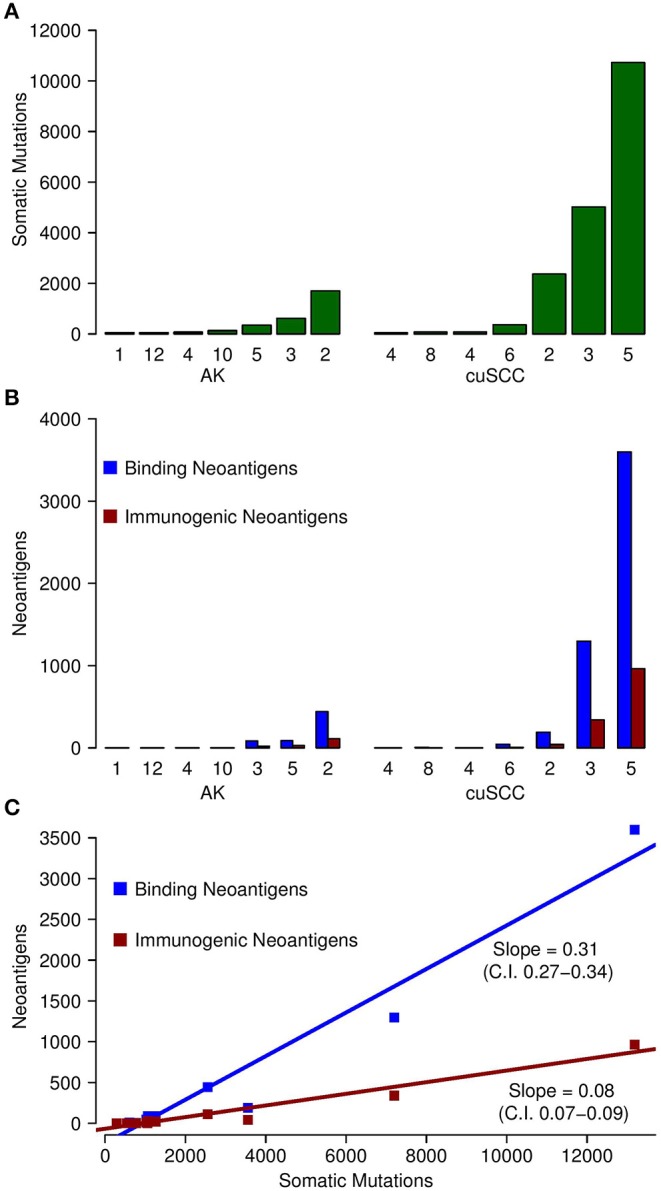
**(A)** Comparison of the number of somatic mutations in each of the samples for AK and cuSCC. **(B)** In blue, comparison of the number of neoantigens predicted to have an MHC class I dissociation constant of <500 nM (termed Binding Neoantigens) in AK and cuSCC. In red, comparison of the number of neoantigens predicted to have an MHC class I dissociation constant of <500 nM and a non-zero TCR recognition potential (termed Immunogenic Neoantigens) in AK and cuSCC. **(C)** Correlation between the number of somatic mutations and the number of binding neoantigens (blue) and immunogenic neoantigens (red). Each data point represents an AK or cuSCC sample. Numbers for AK and cuSCC indicate the patient sample from Table 1 used for the analyses. Note that the AK and cuSCC samples from an individual patient are separate lesions.

Somatic mutations and neoantigens were identified and filtered using the methods described. As previously shown by Chitsazzadeh et al. the mutational burden varied widely over both the AK and cuSCC samples (19). For cuSCCs there was an average of 2,861 mutations with a range of 389–11,504 and for AKs there was an average of 424 mutations with a range of 346–1,697 (Table 2, Figure 1A). While the maximum number of mutations was higher for the cuSCC samples than the AK samples, there was no statistically significant difference in the average number of mutations (*p* = 0.3379).

**Table 2:** Summary of the number of somatic mutations, 21 base pair peptides (predicted from only non-synonymous mutations), 9 base pair binding neoantigens, and immunogenic neoantigens for each patient sample.

**Sample**	**Somatic mutations**	**Peptides (21 mer)**	**Possible neoantigens (9 mer)**	**Binding neoantigens**	**Immunogenic neoantigens**
Patient 1 AK	50	2	26	0	0
Patient 2 AK	1,697	1,191	15,480	442	112
Patient 2 cuSCC	2,540	2,185	28,403	190	41
Patient 3 AK	617	317	4,121	85	21
Patient 3 cuSCC	5,385	2,941	38,231	1,297	339
Patient 4 AK	75	2	27	2	0
Patient 4 cuSCC1	83	12	156	2	1
Patient 4 cuSCC2	47	1	13	0	0
Patient 5 AK	346	197	2,561	88	28
Patient 5 cuSCC	11,504	7,678	99,799	3,600	965
Patient 6 cuSCC	389	245	3,185	45	8
Patient 8 cuSCC	82	12	157	7	0
Patient 10 AK	133	24	312	2	0
Patient 12 AK	53	0	0	0	0

*Binding neoantigens are those neoantigens with <500 nM dissociation constant from MHC class I, and immunogenic neoantigens are those neoantigens with a non-zero TCR-binding potential*.

The number of binding neoantigens was defined as those neoantigens predicted by NetMHCpan to have a dissociation constant for MHC class I of <500 nM. AK and cuSCC samples both had large ranges of binding neoantigens (88–442 and 45–3,600, respectively), but there was no statistically significant difference in the average number observed for the two populations (Table 2, Figure 1B, *p* = 0.336). These data show that neoantigens predicted to bind MHC class I are present in both AK and cuSCC samples.

The number of neoantigens can be further refined by selecting only those with a non-zero predicted TCR recognition potential. We will refer to these as “immunogenic neoantigens.” Again, a large range was observed (21–112 for AKs, 8–965 for cuSCCs), but no statistically significant difference was present (Table 2, Figure 1B, *p* = 0.3185). These data show that the number of neoantigens can be further refined by incorporating predicted T cell recognition to yield a smaller subset of neoantigens, which are predicted to be immunogenic.

As shown in Figure 1C, there was a strong correlation between the somatic mutations and both the binding and immunogenic neoantigens (Spearman's rho correlation coefficients of 96 and 92%, respectively). However, these correlations represent different associations. The slope of the somatic mutation to binding neoantigen correlation was 0.31 (95% CI of 0.27–0.34), and the slope of the somatic mutation to immunogenic neoantigen correlation was 0.08 (95% CI of 0.07–0.09). Given that there was no overlap for the 95% confidence intervals, we can conclude that the difference in these slopes was statistically significant. While both the number of binding and immunogenic neoantigens increase with an increase in the number of somatic mutations, the increase in the number of immunogenic neoantigens is less than the increase in the number of binding neoantigens. These data shown that the immunogenic neoantigens are a much smaller subset than those with predicted MHC binding capacity.

### Analysis of Neoantigen Fitness Cost

To continue our comparison of the neoantigens present in AKs compared to those in cuSCCs, we analyzed only those samples with more than one immunogenic neoantigen (AKs from patients 2, 3, and 5 and cuSCCs from patients 2, 3, 5, and 6) as this enabled us to look at the comparative strengths of the neoantigens in each population. Note that patient 4 had one immunogenic neoantigen in one of the cuSCC samples. However, this patient was not included in the subsequent analyses as the TCR binding and fitness cost of this neoantigen were 1.77 × 10^−4^ and 2.36 × 10^−5^, respectively. These values were 2 orders of magnitude smaller than the averages for all the other samples included, and thus, this sample was excluded.

**Figure 2:**
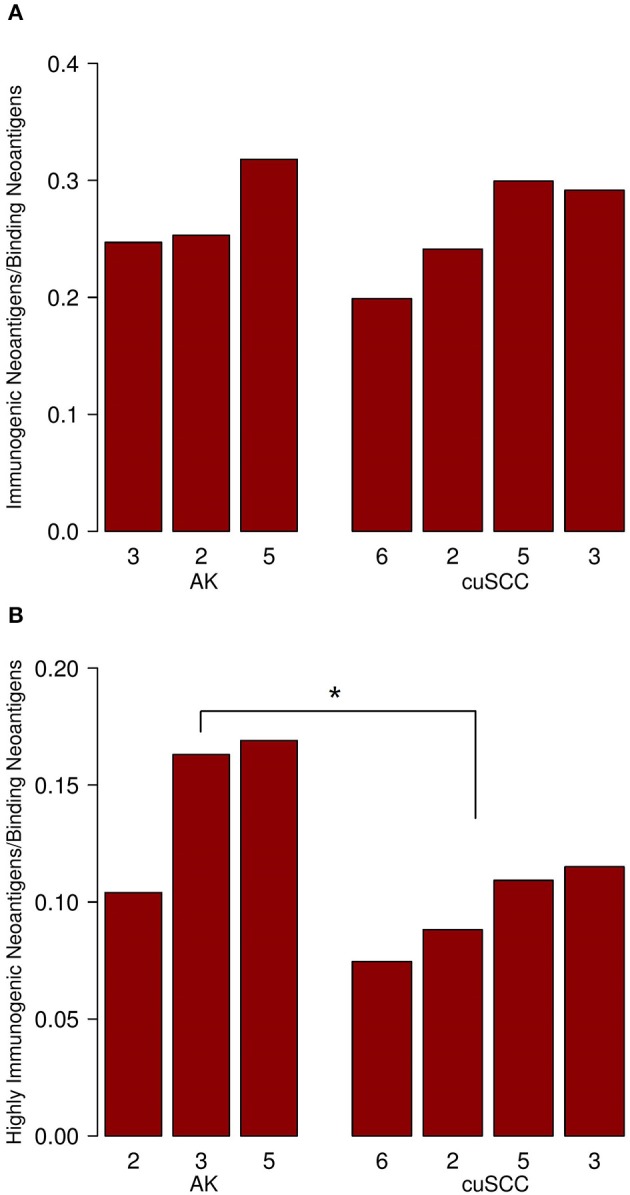
**(A)** Ratio of immunogenic neoantigens to binding neoantigens for AK and cuSCC. **(B)** Ratio of those immunogenic neoantigens with a TCR recognition potential of ≥0.01 to the binding neoantigens. Numbers for AK and cuSCC indicate the patient sample from Table 1 used for the analyses. *p < 0.05.

To examine the percent of neoantigens recognized by the immune system in the AK and cuSCC samples comparatively, we took the ratio of the number of immunogenic neoantigens to the number of binding neoantigens. The ratios were similar for both AKs and cuSCCs (averages of 0.273 and 0.231, respectively, Figure 2A). However, if a more stringent criteria for the binding of the TCR was applied where the binding to the TCR must be ≥0.01, the average for AKs was 0.0290 and the average for the cuSCCs was 0.0147, which was a statistically significant difference (*p* = 0.033, Figure 2B). Since a greater percentage of the mutations in AKs are theoretically visible to the immune system, there may be greater immune recognition of these lesions despite the lack of difference in their mutational rates.

**Figure 3:**
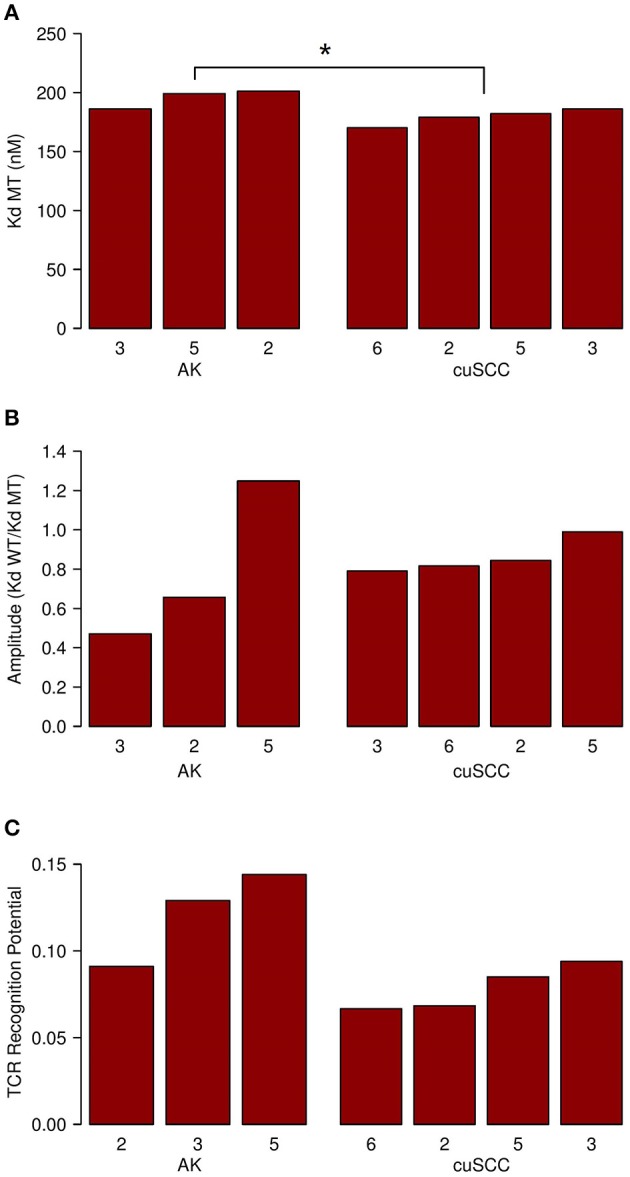
**(A)** Average dissociation constant (K_d_) for mutant (MT) peptide:MHC in AK and cuSCC. **(B)** Average amplitude of MHC binding calculated as the ratio of the dissociation constant (K_d_) for wild-type (WT) peptide:MHC to the dissociation constant (K_d_) of the mutant (MT) peptide:MHC in AK and cuSCC. **(C)** Average TCR recognition potential for AK and cuSCC. Numbers for AK and cuSCC indicate the patient sample from Table 1 used for the analyses. *p < 0.05.

Next, we considered the relative MHC class I binding potential for the neoantigens in AKs compared to cuSCCs. The AK samples had a slightly higher average MHC class I dissociation constant (195 nM) than did the cuSCCs (179 nM), which was statistically significant (Figure 3A, *p* = 0.0497). This indicates that, on average, the neoantigens from AKs were predicted to bind MHC class I with lower affinity than those from cuSCC. Considering this factor alone, it would seem to suggest that the AKs should be less visible to the immune system than the cuSCCs. However, as T cells that recognize self-antigens are eliminated during T cell development, Łuksza et al. propose that if the binding of the wild-type peptide is high, it is likely that the T cell that would recognize this antigen has already been deleted (15). To adjust for this, the amplitude was calculated as the ratio of the dissociation constant for the wild-type peptide:MHC to the dissociation constant for the mutant peptide:MHC. As explained by Łuksza et al., the amplitude reflects the relative probability that a neoantigen will be bound to MHC class I times the relative probability that the corresponding wild-type peptide will not be bound (15). In all cases except AK #5, the amplitude is <1.0, indicating that on average the wild-type peptides were predicted to bind MHC class I with greater affinity that the mutated peptides or neoantigens. The average amplitudes for AK and cuSCC were similar (Figure 3B, *p* = 0.4795), suggesting that any difference in the MHC binding potential of the two groups is eliminated when the relative binding of the wild-type peptide is accounted for. Finally, the average TCR recognition potential was 0.121 for AK and 0.078 for cuSCC (Figure 3C, *p* = 0.0771). While the trend is toward a higher TCR recognition potential for AK than cuSCC, at our sample size this variable is unable to definitively differentiate the two groups.

**Figure 4:**
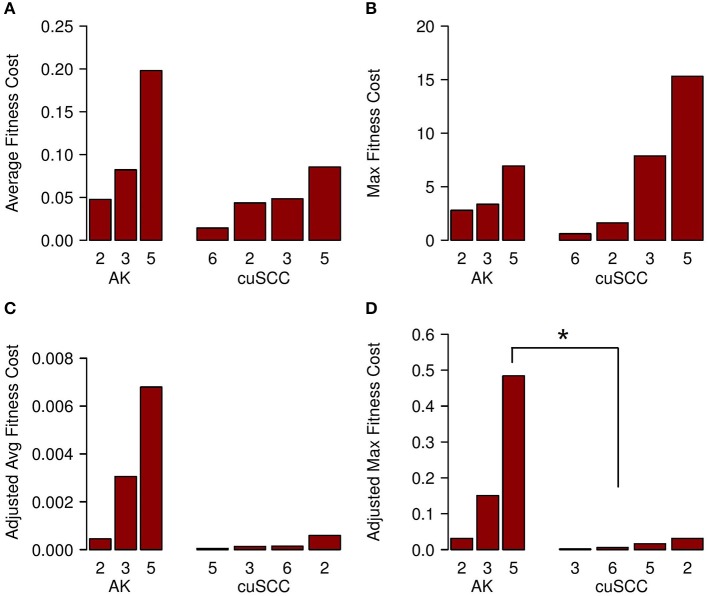
**(A)** Average fitness cost for AK compared to cuSCC. Fitness cost is defined as the MHC-binding amplitude multiplied by the TCR recognition potential. **(B)** Maximum (Max) fitness cost for AK compared to cuSCC. Maximum fitness cost is defined as the highest calculated fitness cost across all neoantigens for each lesion. **(C)** Adjusted average (Avg) fitness cost for AK compared to cuSCC. Adjusted fitness cost is defined as the MHC-binding amplitude multiplied by the TCR recognition potential multiplied by the ratio of RPKM expression for the individual neoantigen to the sum of the RPKM expression of all neoantigens. **(D)** Maximum adjusted fitness cost for AK compared to cuSCC. Maximum adjusted fitness cost is defined as the highest calculated fitness cost after adjustment for RNA expression. Numbers for AK and cuSCC indicate the patient sample from Table 1 used for the analyses. *p < 0.05.

The fitness cost of the neoantigens was defined as the amplitude times the TCR recognition potential. The fitness cost predicts the immune recognition potential of the neoantigen, such that an increased fitness cost is predicted to have greater immune-mediated destruction. The average fitness cost for AK was 0.109, while the average for the cuSCC was 0.048; this difference was not statistically significant (*p* = 0.288, Figure 4A). However, given that T cell-mediated immune responses are mounted to one or a few “immunodominant” antigenic peptides, another important factor to consider is the neoantigen with the highest fitness cost. The maximum fitness cost had a very large range for the cuSCCs (0.604–15.309) compared to a smaller range (2.791–6.941) for AKs (Figure 4B). Despite the lack of statistically significant difference between the maximum fitness costs of AKs and cuSCCs (*p* = 1.0), it is notable that some of the cuSCCs had neoantigens with a greater fitness cost than those of the AKs.

In an attempt to explain why some cuSCCs had neoantigens with a high fitness cost, suggesting that these cuSCCs had neoantigens with high immune recognition potential yet escaped immune-mediated destruction, we adjusted the fitness cost values based on the expression of the neoantigens using the RPKM-normalized RNAseq data. There was a trend for an increased adjusted average fitness cost in AKs (0.00344) compared with cuSCCs (0.00023) (Figure 4C, *p* = 0.08). When evaluating the neoantigen with the maximum fitness cost after adjusting for expression, adjusted maximum fitness cost for AKs (0.22) was significantly increased compared with cuSCCs (0.014) (Figure 4D, *p* = 0.03). Taken together these data demonstrate that while cuSCCs have neoantigens with immune recognition potential, these neoantigens are not expressed, resulting in a higher adjusted maximum fitness cost in AKs compared to cuSCC.

### Analysis of HLA Mutations and Immune Infiltration

We analyzed HLA-A, B, and C for both mutations and expression levels because recognition of neoantigens by the immune system requires functional MHC class I proteins to be made and expressed within the cell. Mutational analysis revealed two somatic mutations within HLA coding regions for the HLA-B gene in the cuSCC sample from patient 2 and the HLA-C gene in the AK sample from patient 5. However, neither of these mutations were within the peptide binding groove, which suggests that these mutations do not alter peptide binding. Additional analysis was performed to examine whether the mutations affected the expression level. Expression of HLA-B for patient 2 cuSCC was within one standard deviation of the mean expression and expression of HLA-C for patient 5 AK was within two standard deviations of the mean, suggesting that these mutations do not alter expression. Mutations in members of the MHC class I pathway (TAP1, TAP2, and B2M) can also lead to the loss of cell surface MHC class I expression and immune escape (39). Therefore, we interrogated TAP1, TAP2, and B2M genes for mutations, but no mutations in these genes were identified. We used a linear regression analysis to determine if the rate of expression of HLA (A, B, and C), TAP1, TAP2, or B2M correlated with the adjusted maximum fitness cost. No significant correlation was found between the adjusted maximum fitness cost and the normalized expression of any of the six proteins analyzed (data not shown). This result suggests that there was no meaningful impact of mutations in or expression of these proteins on the fitness cost of the tumor.

Analysis was also done to compare the level of immune infiltration (as determined by xCell analysis) to the neoantigen strength. Linear regressions were fit to the adjusted maximum fitness cost against the expression level of each of the following: T cell subsets (CD4 T cells, CD8 T cells, CD8 central memory T cells, CD8 effector memory T cells, CD8 naïve T cells, regulatory T cells), dendritic cell populations (immature DCs and conventional DC) and NK cells. There were trends for increased CD4 and CD8 T cell signatures in the tumor with increased adjusted maximum fitness cost (data not shown). However, no statistically significant associations were identified, which may in part be due to the small sample size.

## Discussion

Given that a low percentage of AKs progress to cuSCCs, it is important to understand what allows some of these precursor lesions to escape immune surveillance and become invasive cuSCC, while others AKs remain in equilibrium or are eliminated. An improved understanding of immunoediting in AKs and cuSCCs may aid clinical decision making and assist the development of preventive or treatment strategies. Chitsazzadeh et al. found, and we confirmed, that the maximum number of somatic mutations is greater in the cuSCCs than the AKs (though the large range prevents a statistically significant difference in the average number) (19). This finding is also consistent with work showing that esophageal adenocarcinomas have higher rates of mutations than the precursor Barrett's esophagus lesions (17, 18). To our knowledge, our study is the first to identify neoantigens in AKs and cuSCCs and compare the quality of neoantigens between precursor and invasive, cancerous lesions. A limitation of our analyses was the small sample size; unfortunately, no other publicly-accessible WES and RNAseq datasets were found for AK and cuSCC samples in PubMed, NCBI dbGaP or Google Scholar. We identified binding neoantigens (based on predicted MHC binding) and immunogenic neoantigens (based on predicted MHC binding and TCR recognition potential) in both AKs and cuSCC with similar findings to the number of somatic mutations. The maximum number of both binding and immunogenic neoantigens is higher in cuSCCs than AKs, but there was not a statistically significant difference in the average number. These findings indicate that there must be some factor other than the number of mutations and neoantigens that influences the immune-evasion potential of the tumor.

There was variation in the number of mutations and neoantigens across the different samples. This magnitude of variation is consistent with a prior study that has shown large differences in somatic mutations even in those tumors with high mutational burdens (40). Our results are also consistent with previous work reporting a small number of mutations that form binding neoantigens (41). For a mutation to lead to a neoantigen it must be in a coding region of the DNA, change the amino acid sequence, and meaningfully bind the MHC molecule. Because of these criteria, only a small number of the mutations accumulated in a tumor will create binding neoantigens and an even smaller number of these will be able to be recognized by the immune system as immunogenic neoantigens (as is reflected in Table 2).

One significant difference observed between the cuSCCs and the AKs is the percentage of their neoantigens that are highly recognized by TCRs. The cuSCCs on average had a lower percentage of binding neoantigens that are also predicted to be highly recognized by the TCRs. Thus, the presence of a greater numbers of neoantigens in cuSCCs may not directly correlate to the visibility of that lesion to the immune system. Rather, the increased percentage of neoantigens that are recognized in AKs compared to cuSCCs indicates that the TCR recognition of the neoantigens predicted to bind MHC may be an important factor in determining the success of immunosurveillance. This finding corroborates the work of Anagnostou et al. who demonstrated that, after immunotherapy, the number of neoantigens increased, but the strength of those neoantigens decreased (16).

When considered separately, neither predicted MHC binding nor predicted TCR recognition of neoantigens appears to account for a difference in immune evasion of cuSCC compared with AK. The predicted affinity of neoantigens binding MHC in cuSCC is slightly higher on average than in AK, which would suggest that the immune system should be able to recognize the cuSCCs more readily. However, this difference is eliminated when the relative binding of the corresponding wild-type peptide is considered. This suggests that the negative selection of self-reactive T cells is one important factor to consider in determining the ability of the immune system to recognize the tumors. We observed a trend for a difference in TCR recognition potential between AKs and cuSCCs (*p* = 0.0771), suggesting that, perhaps, with a larger sample size, this might prove to be a statistically significant factor in determining immune recognition. However, with our current sample size, it is not possible to determine if TCR recognition potential alone is able to explain the difference in immune evasion between the pre-cancerous and cancerous lesions.

Predicted MHC binding and TCR recognition potential were then combined into an overall fitness cost, as has been done by Łuksza et al. (15), but the average and maximum fitness costs were found to have no statistically significant difference between AKs and cuSCCs. Surprisingly, the range of the maximum fitness costs included higher values for the cuSCCs than the AKs. This result seemed to contradict the expected result that the cuSCCs should have enhanced immune evasion mechanisms than the AKs, as they have escaped immune recognition to develop into invasive cancers. We then incorporated RNAseq expression data into our calculation since a neoantigen of any strength cannot be recognized by the immune system unless it is appreciably expressed by the cell. When the expression data was incorporated, the maximum fitness cost for AKs was significantly increased compared with cuSCCs, and there was a trend for increased average fitness cost for AKs compared with cuSCCs. These results suggest that, although the cuSCCs accumulate many potentially immunogenic neoantigens (many of which are stronger than the immunogenic neoantigens of the AKs), these neoantigens are not appreciably expressed. This leads us to hypothesize that down-regulating the expression of these strong neoantigens is a mechanism of immune evasion for cuSCCs that may allow them to escape the immune system whereas the AKs fail to do so. Consistent with this hypothesis, Rosenthal et al. demonstrated that there are higher rates of gene suppression in those genes with mutations that formed neoantigens compared to genes with non-neoantigenic mutations (7). The work of Matsushita et al. also corroborates our finding by showing that a strong neoantigen is downregulated in those tumors which succeed in growing in an immunocompromised host compared to an immunodeficient host (42). Their work also corroborates the suggestion that it is the maximum strength neoantigen that is of most importance in determining the immune detection of the tumor.

By identifying and comparing the quality of neoantigens in AKs and cuSCCs, we show that cuSCCs have lower rates of predicted TCR recognition of neoantigens that bind MHC class I than do AKs. Additionally, when expression is considered, many of the strong neoantigens from cuSCCs are insufficiently expressed, causing the maximum fitness cost (and by extension, immune recognition) to be lower for cuSCCs than for AKs. Our findings shed light on why tumor mutational burden alone may not accurately predict the anti-tumor immune response in treatment with immune checkpoint inhibition (9–13). Our results also suggest a role for the down-regulation of highly immunogenic neoantigens in the escape of cuSCCs from immune recognition.

## Data Availability Statement

The RNAseq data analyzed in this study was obtained from NCBI/GEO with SuperSeries accession code GSE84194 (https://www.ncbi.nlm.nih.gov/geo/query/acc.cgi?acc=GSE84194) and the WES data was obtained from Dr. Kenneth Tsai. Requests to access the WES datasets should be directed to Dr. Kenneth Tsai, kenneth.tsai@moffitt.org.

## Author Contributions

EB, KB, and KH contributed conception and design of the study. EB performed the calculations and wrote the first draft of the manuscript. PK completed the statistical calculations. HN, TP, and MW provided the neoantigen identification pipeline and technical assistance in running the calculations. All authors contributed to manuscript revision, read and approved the submitted version.

### Conflict of Interest

The authors declare that the research was conducted in the absence of any commercial or financial relationships that could be construed as a potential conflict of interest.
